# Vitamin E Analogue Improves Rabbit Sperm Quality during the Process of Cryopreservation through Its Antioxidative Action

**DOI:** 10.1371/journal.pone.0145383

**Published:** 2015-12-23

**Authors:** Zhendong Zhu, Xiaoteng Fan, Yinghua Lv, Nan Zhang, Chuning Fan, Pengfei Zhang, Wenxian Zeng

**Affiliations:** College of Animal Science and Technology, Northwest A&F University, Shaanxi, 712100, China; Medical College of Georgia, UNITED STATES

## Abstract

The process of cryopreservation results in high concentration of reactive oxygen species which is detrimental to spermatozoa. The aim of this study was to investigate whether addition of vitamin E analogue to freezing extender can facilitate the cryosurvival of spermatozoa in rabbits, and how vitamin E protects spermatozoa against damages during the process of preservation. Freshly ejaculated semen was diluted with Tris-citrate-glucose extender supplemented with different concentrations of Trolox (a vitamin E analogue). The level of radical oxygen species (ROS) in spermatozoa that was exposed to Trolox was significantly lower than that of the control during each step of the process of preservation. The percentage of frozen-thawed spermatozoa with lipid peroxidation in the Trolox treatments was significantly lower than that of the control. The motility, intact acrosome, membrane integrity and mitochondrial potentials of the frozen-thawed spermatozoa in the treatment of 200 μM Trolox were significantly higher than those of the control. These observations suggest that addition of vitamin E to a freezing extender leads to higher integrity of acrosome, plasma membrane and mitochondrial membrane potential as well as higher motility. Vitamin E protects spermatozoa through its capacity to quench ROS accumulation and lipid peroxidation during the process of preservation. Addition of Trolox is recommended to facilitate the improvement of semen preservation for the rabbit breeding industry.

## Introduction

Cryopreservation of semen has been of great benefit to agriculture, conservation of wild animals and treatment of human infertility [1]. However, frozen-thawed spermatozoa have not been widely applied in the rabbit industry due to the low quality and fertility of spermatozoa and small litter sizes [2]. The freezing-thawing process results in high concentrations of reactive oxygen species (ROS) [3–4] which are detrimental to spermatozoa as the plasma membranes are rich in long-chain polyunsaturated fatty acids [5]. Free radical may lead to lipid peroxidation (LPO) which is accompanied by extensive structural alterations, particularly in the acrosomal region, a rapid and irreversible loss of motility, a profound change in metabolism, and a high leakage rate of intracellular cell constituents [6].

As a result, addition of antioxidants to freezing extenders may help spermatozoa against oxidative stress. Supplementation of vitamin E with cryopreservation media prevented oxidative damage to spermatozoa and improved cryosurvival in boars [7–10], bulls [11–12], buffalo bulls [13], humans [14–15], rams [16–18], and sheep bucks [19]. However, it is unknown whether vitamin E protects spermatozoa from cryo-damage via its capacity to quench ROS. Meanwhile, recent reports showed that vitamin E presented contradictory results in red deers [20] and goat bucks [21]. In addition, catalase and vitamin E analogue were presented in the seminal plasma of rabbits [22–24]. Therefore, the aim of this study was to investigate whether addition of vitamin E analogue to freezing extender can facilitate the cryosurvival of spermatozoa in rabbits, and how vitamin E protects spermatozoa against damages during the process of preservation.

## Materials and Methods

### Extender preparation

The basic extender was a Tris-citrate-glucose extender (TCG), composed of 250mM Tris-hydroxymethylaminomethane, 87.5mM citric acid, 69mM glucose, 100 million IU penicillin sodium and 100million IU streptomycin sulphate, pH 6.8. The freezing extenders were TCG supplemented with 20% (v/v) egg yolk, 4% (v/v) DMSO (final concentration) and vitamin E analogue (Trolox). The final concentrations of Trolox in TCG extender were 0, 100, 200, 250 μM.

### Animals

All experimental procedures involving animals were approved by the Northwest A&F University’s Institutional Animal Care and Use Committee. Five 1-year-old male rabbits (body weights 3.0 kg–4.5 kg) were used in this study. Each rabbit was put in a single cage with a photoperiod of 16 / 8 h light/day, fed with a commercial standard diet, and allowed free access to water. Two mature female rabbits were used as teasers for collection of semen.

### Semen processing

Semen was collected using an artificial vagina on a regular basis (two collections per week). Ejaculates were pooled together to avoid individual differences. The semen were incubated in pre-warmed water and delivered to the laboratory within an hour for evaluation. The semen with over 90% of motile spermatozoa was mixed.

The freshly collected semen was diluted with TCG at room temperature, and gradually cooled to 5°C within 2 h. The semen was diluted with the freezing extenders (v:v = 1:1) and kept for 30 min at 5°C. The diluted semen was packed into 0.25 mL- straws immediately (3×10^7^ spermatozoa per straw). The straws were placed horizontally 5 cm above the surface of liquid nitrogen for 10 min, and plunged into liquid nitrogen. After stored in the liquid nitrogen for at least one week, the frozen semen was thawed in a water bath at 37°C for 30 s.

At least 10 straws for each group were frozen from each collection of semen. Each time, we thawed two straws of semen from each treatment group for an evaluation of a sperm parameter. It took less than 10 min between the first straw thaw and the last straw thaw. The control and Trolox treatments were replicated 3 times with 3 different collections of semen. Each collection of semen represented 1 replicate.

### Sperm motility

Two straws of frozen semen from each experiment group were transferred into a water bath at 37°C for 30 s. The thawed-semen was pooled together as one sample, diluted with TCG extender (1:4) and incubated in a water bath at 37°C for 15 min. Sperm motility was evaluated by visual estimation. A drop of 10 μL of sperm suspension was delivered onto a pre-warmed clean glass slide, and covered with a clean coverslip. The slides were examined under an optical microscope with a bright field (Nikon 80i; Tokyo, Japan) at 200× total magnification. The percentages of sperm showing progressive movement were estimated and noted after viewing five different fields. Three separate aliquots (replicates) were assessed from each semen sample.

### Sperm membrane integrity

SYBR-14 and propidium iodide (Sperm Viability Kit Molecular Probes, Leiden the Netherlands, L7011) were used to evaluate the membrane integrity [25]. Briefly, aliquots of 20 μL sperm supernatant were added to 100 μL of HEPES buffered saline solution (10 mM HEPES, 150 mM NaCl, 10% BSA, pH 7.4), and stained with 0.12 μL of the SYBR-14 working solution (100 nM in DMSO) for 10 min at 36°C in the dark, followed by addition of 0.6 μL of propidium iodide (PI) stocking solution (2.4 mM in water), then incubated for additional 10 min.

Sperm staining was monitored and photographed by an epifluoresence microscope (Nikon 80i; Tokyo, Japan) with a set of filters (400X) with 535 nm excitation and 617 nm emission for PI red fluorescence and 488 nm excitation and 516 nm emission for SYBR-14 green fluorescence. For the same field, photographs were also taken with a phase-contrast microscope. At least 200 spermatozoa per slide were counted. Three separate aliquots (replicates) were assessed from each semen sample. All samples were barcoded and evaluated by one observer.

### Acrosomal intact

For the acrosome staining, a protocol described by Zeng et al (2001) [26] was used after a slight modification. Briefly, 30 μL of the sperm samples were smeared onto a clean glass slide, air-dried, then fixed with absolute methanol for 10 min. Then, additional 30 μL of fluorescein isothiocyanate-peanut agglutinin (FITC-PNA, Sigma) solution (100 μg/mL) dilutes in PBS were spread over each slide. The slides were incubated in a dark and moist chamber at 37°C for 30 min, and subsequently washed with PBS twice and air-dried in the dark. Ten microliters of antifade solution (P0123; Beyotime, China) was added to the slide to preserve fluorescence before a clean coverslip was applied. The edges of the coverslip were sealed with colorless nail polish.

The acrosomal status of the spermatozoa was monitored and photographed by an epifluoresence microscope (Nikon 80i; Tokyo, Japan) with a set of filters (400X) with 488 nm excitation and 525 nm emission. For the same field, photographs were also taken with a phase-contrast microscope. At least 200 spermatozoa per slide were counted. Three separate aliquots (replicates) were assessed from each semen sample. All samples were barcoded and evaluated by one observer.

### Mitochondrial membrane potentials (ψm)

The changes of sperm mitochondrial membrane potential (ΔΨm) were evaluated using a JC-1 (lipophilic cation 5,5^’^, 6,6’- tetrachloro-1,1’,3,3’ -tetraethylbenzimidazolcarbocyanine iodide) Mitochondrial Membrane Potential Detection Kit (Beyotime Institute of Biotechnology, China), following the manufacturer’s instruction [27–28]. Briefly, the sperm samples (2×10^6^/mL) were stained with 28 μL of JC-1 (stock solution) in PBS (final volume, 100 μL). After incubation at 37°C for 30 min in the dark‚the samples were centrifuged at 600 x g for 5 min, and re-suspended in JC-1 buffer and placed on ice. Sperm samples were immediately analyzed using a flow cytometer (FAC SCalibur, BD Biosciences) with excitation at 525 nm and emission at 590 nm. A total of 20,000 sperm-specific events were evaluated and calculated as percentages. Data were processed using the CellQuest program (BD Biosciences).

### Lipid peroxidation

As in previous reports, BODIPY 581/591C_11_ (Molecular Probes), a sensitive fluorescent probe for lipid peroxidation (LPO), was used to measure lipid peroxidation [29–30]. Briefly, 200 μL of sperm samples were washed twice by centrifugation for 5 min at 200×g. The BODIPY 581/591C_11_ probe (working solution: 10 μM) was added to the re-suspended sperm suspensions to bring the probe to a final concentration of 2 μM, and incubated at 37°C for 30 min in the dark. The samples were washed by centrifugation at 800×g for 5 min to remove the unbound probe, and analyzed with a flow cytometer (FACSCalibur, BD Biosciences). Red fluorescence was measured using an FL2 longpass filter (>670 nm). A total of 20,000 sperm-specific events were evaluated and calculated as percentages. Data were processed by using the CellQuest program (BD Biosciences).

### Measurement of intracellular reactive oxygen species

Reactive Oxygen Species Assay Kit (Beyotime Institute of Biotechnology, China) was applied to measure the level of intracellular reactive oxygen species following the manufacturer’s instruction. DCFH-DA is oxidized by reactive oxygen species to 2′, 7′-dichlorofluorescein (DCF) which is highly fluorescent at 530 nm. Briefly, sperm samples were washed three times with TCG extender. Sperm sediments were suspended with TCG extender containing 10 μM DCFH-DA (10x10^6^ spermatozoa /ml), and incubated for 30 min at 37°C in the dark. The samples were washed three times with TCG extender. The relative levels of fluorescence were quantified by a multi-detection microplate reader (485 nm excitation and 535 nm emission).

### Experiment design

Experiment I was designed to detect whether addition of the vitamin analogue Trolox to freezing extender could improve the quality of frozen-thawed rabbit spermatozoa via examination of sperm motility, plasma membrane integrity, intact acrosome, mitochondrial membrane potential and lipid peroxidation. Experiment II was set up to study whether vitamin E analogue Trolox could protect spermatozoa during the process of cryopreservation from cooling, equilibration, freezing and thawing, to incubation of frozen-thawed spermatozoa in vitro. Experiment II was performed to further to reveal whether vitamin E protects spermatozoa through its antioxidative potential. Vitamin E analogue Trolox was supplemented in the TCG extender, freezing extender, and thawing solution. Sperm parameters and ROS level were detected during the whole process.

### Statistical analysis

Data were examined using the Kolmogorov-Smirnov test to determine the distribution. All data were analyzed by one-way ANOVA, and multiple comparisons with least significant difference (LSD) was performed using SPSS version 17.0 for Windows (SPSS Inc., Chicago, IL). All the values are presented as mean ± standard error of the mean (SEM). A probability (p) value of less than 0.05 was considered to be statistically significant.

## Results

### Experiment I

#### Sperm motility

**Fig 1:**
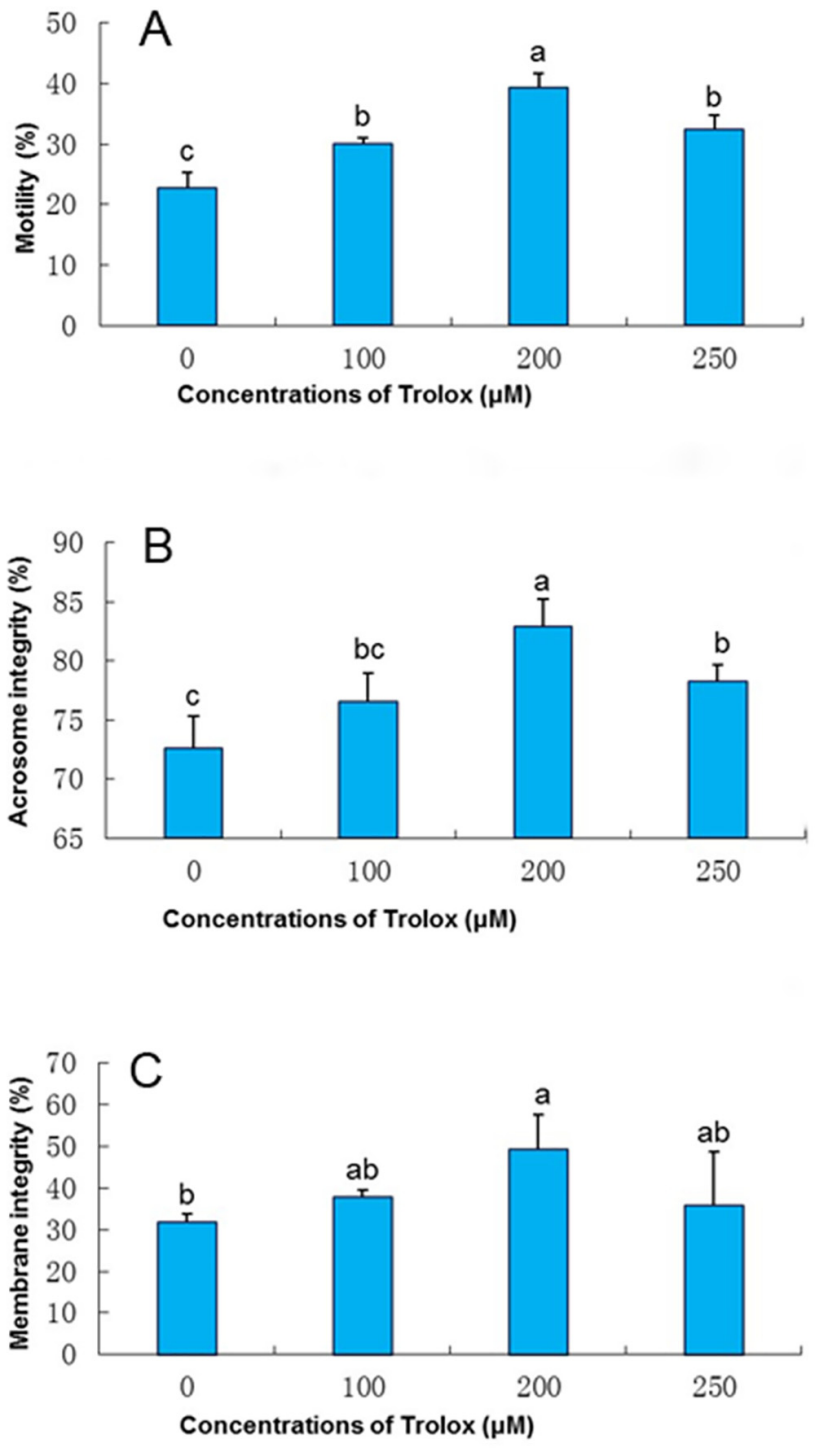
Effect of supplementation TCG extender with Trolox on motility (A), intact acrosomes (B) and membrane integrity (C) of the frozen-thawed rabbit spermatozoa. Bars represent the mean ± SEM (n = 3 independent replicates). Different lower-case letters denote significant differences (P < 0.05).

As shown in Fig 1A, motility of the frozen-thawed spermatozoa in the Trolox treatments was significantly higher than those in the control group (p<0.05). Especially, sperm motility in the treatment supplemented with 200 μM of Trolox in freezing extender was the highest among them, significantly higher than those of 100 μM or 250 μM Trolox treatment. These observations suggest that addition of Trolox to the freezing extender could improve the motility of the frozen-thawed rabbit spermatozoa.

#### Acrosomal status

**Fig 2:**
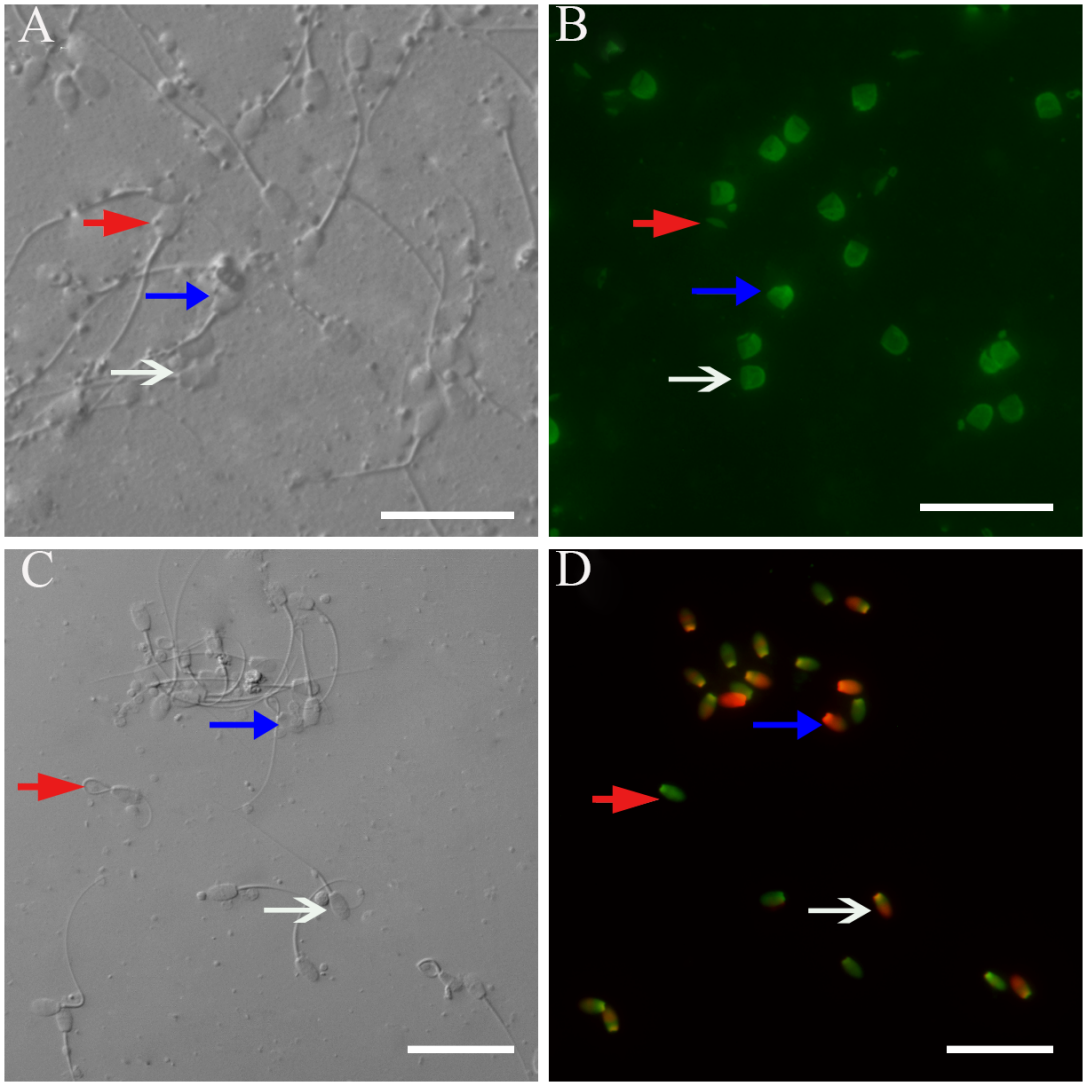
Photomicrographs of the post-thaw rabbit spermatozoa. (A, C) Images obtained by phase contrast microscope. (A) Image obtained by FITC-labeled peanut agglutinin staining, Images (A) and (B) were from the same field. White arrow indicates intact acrosomes; blue arrow indicates partially damaged acrosome; red arrow indicates damaged acrosome. (D) Image obtained by SYBR-14 plus propidium ioide (PI) staining. Images (C) and (D) were from the same field. red arrow indicates membrane integrity; blue arrow indicates membrane damaged; white arrow indicates membrane damaged slightly. Scale bars represent 30 μm.

As shown in Fig 2B, the observed fluorescence images of spermatozoa stained with FITC-PNA were classified into 3 groups: Intact acrosome, spermatozoa with intensively bright fluorescence of the acrosomal cap which were indicated by an intact outer acrosomal membrane; Partially damaged acrosome, spermatozoa with disrupted fluorescence of the acrosomal cap which were indicated by partial disruption of the acrosomal membrane; Damaged acrosome, spermatozoa with no fluorescence which were indicated by a complete loss of the outer acrosomal membrane and was determined under a phase contrast illumination system.

Addition of Trolox to the freezing extender had a great beneficial effect on maintenance of intact acrosome in the frozen-thawed spermatozoa (Fig 1B). The frozen-thawed spermatozoa in the treatments supplied with 200 or 250 μM Trolox in freezing extender showed a significantly higher value of intact acrosomes compared with the control. However, the frozen-thawed spermatozoa in the freezing extender supplied 100 μM of Trolox showed no differences for intact acrosome compared with the control. These data indicate that addition of vitamin E analogue in the freezing extender could protect the acrosome from damage during the freezing-thawing process.

#### Membrane integrity

The spermatozoa were classified into three groups: Group A showing membrane integrity, which were stained green with SYBR-14 but not with PI; Group B showing membrane damage, which were stained red with PI but not with SYBR-14; Group C showing slightly damaged membrane, which were stained green-orange with both SYBR-14 and PI (Fig 2D).

As presented in Fig 1C, the percentage of frozen-thawed spermatozoa with intact cell membrane was 49.2% in the treatment of 200 μM of Trolox which was significantly higher than that in the control (P < 0.05). However, addition of 250 μM Trolox to the freezing extender did not yield better results. These observations suggest that supplementation of 200 μM vitamin E analogue with the freezing extender is the optimal concentration in terms of membrane integrity.

#### Mitochondrial membrane potentials

**Fig 3:**
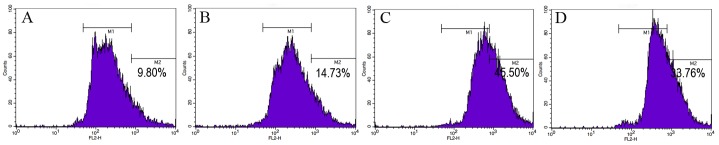
Effect of supplementation TCG extender with Trolox on mitochondrial membrane potential of the frozen-thawed rabbit spermatozoa. Mitochondrial membrane potential was evaluated using JC-1 (lipophilic cation 5,5’, 6,6’- tetrachloro-1,1’,3,3’ -tetraethylbenzimidazolcarbocyanine iodide) Mitochondrial Membrane Potential Detection Kit. Histogram shows spermatozoa with high (marker M2) and low (marker M1) mitochondrial membrane potential. (A: 0 μM; B: 100 μM; C: 200 μM; D: 250 μM).

The value of mitochondrial membrane potentials was significantly increased (P < 0.05) with the addition of vitamin E analogue (Fig 3). Compared to the controls, the red fluorescence intensity was higher in the treatments. The maximum value (45.5%, Fig 3C; M_2_) was noted in spermatozoa supplemented with 200 μM of Trolox, whereas the lowest was in the control treatment (9.8%, Fig 3A; M_2_). Mitochondrial membrane potentials of the other two Trolox treatments (14.7%, Fig 3B; 33.7%, Fig 3D) were also higher than that of the control. The peak of the red fluorescence was achieved with the addition of Trolox, indicating that supplementation of vitamin E analogue leads to an increase of cell mitochondrial membrane potentials.

#### Lipid peroxidation (LPO)

**Fig 4:**
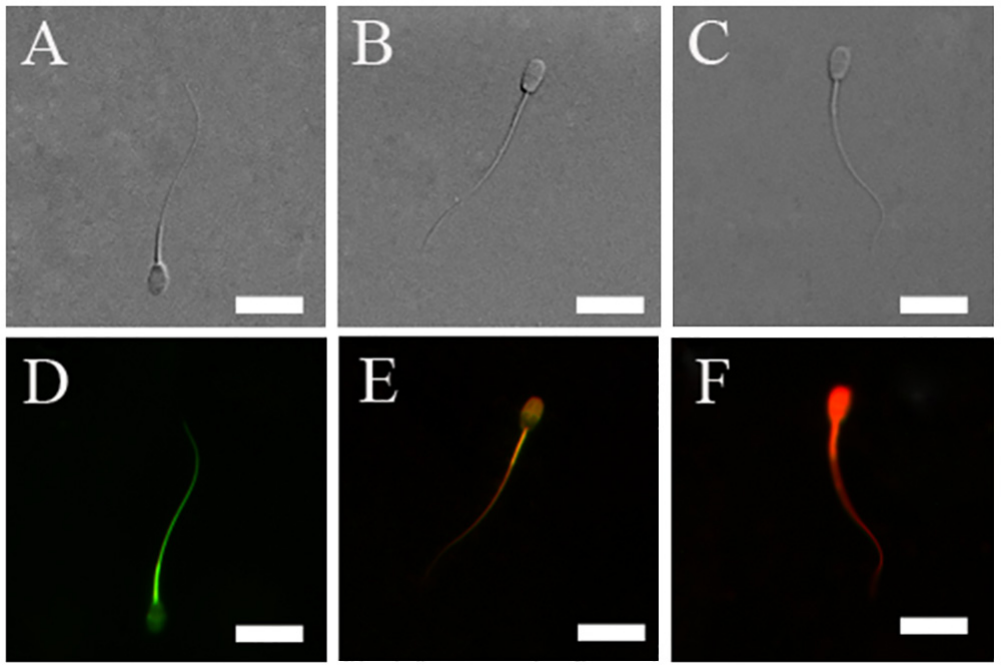
Photomicrographs of rabbit spermatozoa. (A-C) Images obtained using phase contrast microscope; (D-F) Images, which were the same images as (A-C) respectively, obtained by staining with the probe BODIPY 581/591C11. Three kinds of staining could be observed under a fluorescence microscope: (D) indicates that sperm was seriously oxidized; (E) sperm was partly oxidized; (F) sperm was not oxidized. Bars = 15μm.

As showed in (Fig 4D–4F), when spermatozoa were incubated with LPO fluorescent probe BODIPY 581/591C11, three kinds of spermatozoa stained by the probe were observed under the fluorescent microscope. The changes of fluorescence indicated that LPO occurred extensively in the head and mid-piece of the spermatozoa.

**Fig 5:**
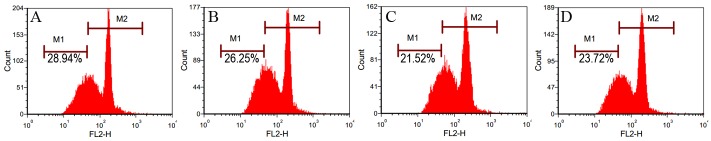
Detection of addition of vitamin E analogue to freezing extender on lipid peroxidation in the frozen-thawed rabbit spermatozoa. Sperm lipid peroxidation was analyzed by staining with BODIPY 581/591C_11_. Histogram shows spermatozoa with lipid peroxidation (marker M1) and without lipid peroxidation (marker M2). A: 0 μM; B: 100 μM; C: 200 μM; D: 250 μM.

In terms of LPO, the percentage of frozen-thawed spermatozoa with LPO was significantly decreased with the addition of Trolox (Fig 5). The minimum value (21.5%) was noted in spermatozoa supplemented with 200 μM Trolox, whereas the highest was in the control treatment (28.9%). The percentages of spermatozoa with LPO in the other two treatments were also lower than the controls. These data suggested that addition of vitamin E analogue protects the spermatozoa from lipid peroxidation.

### Experiment II

#### Sperm parameters

**Fig 6:**
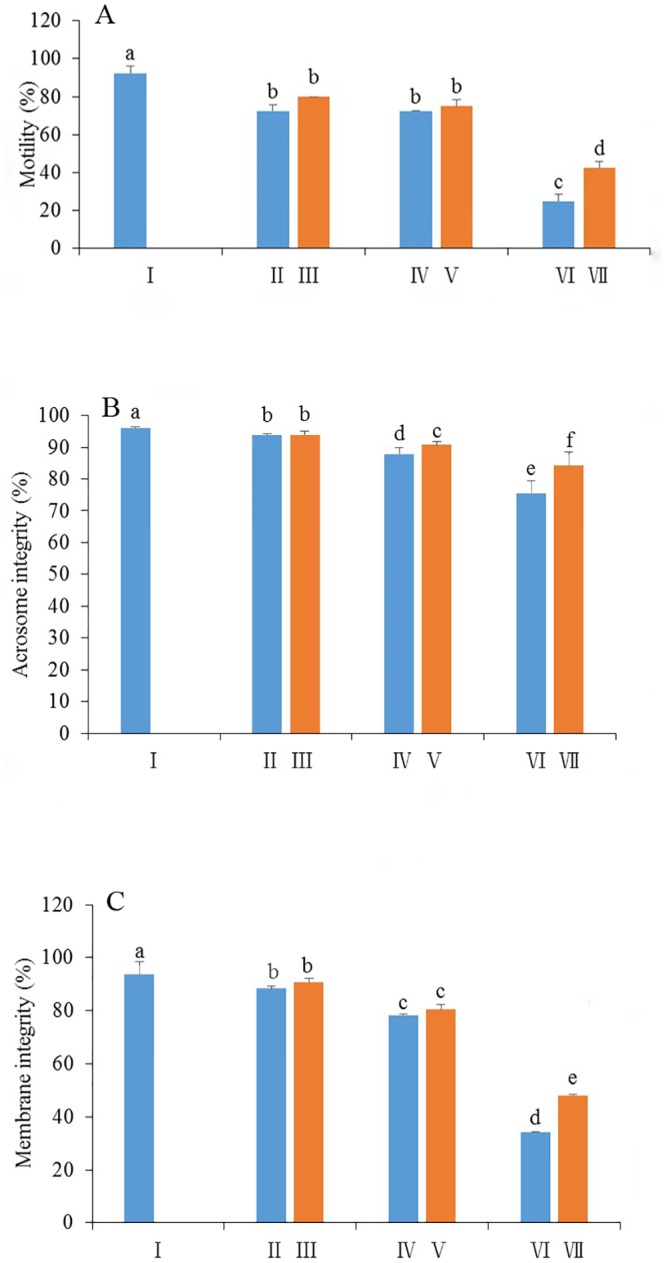
Effect of exposure spermatozoa to vitamin E analogue Trolox on motility (A), acrosome integrity (B), membrane integrity(C) during process of preservation. Bars represent the mean ± SEM (n = 3 independent replicates). Different lower-case letters denote significant differences (P < 0.05). I freshly ejaculated spermatozoa diluted with TCG extender; II freshly ejaculated spermatozoa diluted with TCG extender and cooled from room temperature to 5°C; III freshly ejaculated spermatozoa diluted with TCG extender containing 200 μM Trolox and cooled from room temperature to 5°C; IV freshly ejaculated spermatozoa diluted with TCG extender, cooled to 5°C and equilibrated in freezing extender for 30 min at 5°C; V freshly ejaculated spermatozoa diluted with TCG extender, cooled to 5°C and equilibrated in freezing extender containing 200 μM Trolox for 30 min at 5°C; VI freshly ejaculated spermatozoa diluted with TCG extender, cooled to °C, equilibrated in freezing extender for 30 min at 5°C,frozen and thawed frozen; VII freshly ejaculated spermatozoa diluted with TCG extender, cooled to 5°C, equilibrated in freezing extender containing 200 μM Trolox for 30 min at 5°C, frozen and thawed frozen.

To elucidate whether vitamin E improves sperm parameters at each step of cryopreservation, spermatozoa were exposed to Trolox during the whole process of cryopreservation. As shown in Fig 6, during cooling from room temperature to 5°C and equilibration at 5°C for 30 min, motility, intact acrosome and plasma membrane integrity in spermatozoa exposed to 200 μM Trolox showed a tendency to increase, compared with those without exposure to Trolox. During equilibration at 5°Cfor 30 min, the values of motility and membrane integrity were similar in the two groups. The value of intact acrosome was significantly higher in spermatozoa exposed to Trolox than that without exposure to Trolox (P < 0.05). Interestingly, the values of motility, acrosomal intact and membrane integrity in the frozen-thawed spermatozoa exposure to Trolox were significantly higher than those of spermatozoa without exposure to Trolox, suggesting that exposure of spermatozoa to the vitamin E analogue improves the three sperm parameters during freezing-thawing process.

**Fig 7:**
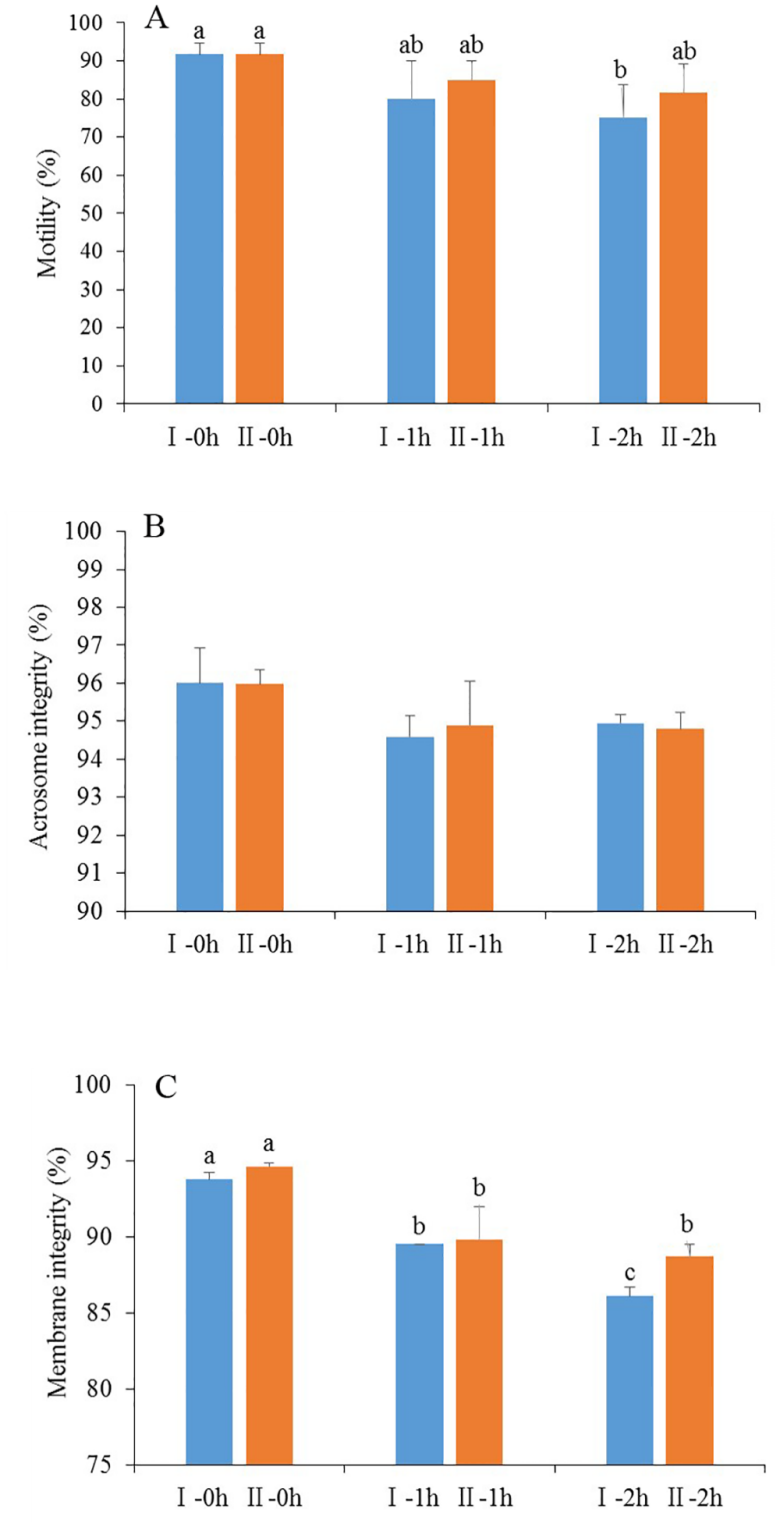
Effects of addition of vitamin E analogue Trolox to TCG extender on sperm motility (A), acrosome integrity (B), membrane integrity (C) during incubation at 37°C for 2 h. Bars represent the mean ± SEM (n = 3 independent replicates). Different lower-case letters denote significant differences (P < 0.05). I-0h fresh spermatozoa incubated in TCG extender without Trolox for 0 h; I-1h fresh spermatzoa incubated in TCG extender without Trolox for 1 h; I-2h fresh spermatozoa incubated in TCG extender without Trolox for 2 h; II-0h fresh spermatozoa incubated in TCG extender containing 200 μM Trolox for 0 h; II-1h fresh sperm incubated in TCG extender containing 200 μM Trolox for 1h; II-2h fresh sperm incubated in TCG extender containing 200 μM Trolox for 2 h.

**Fig 8:**
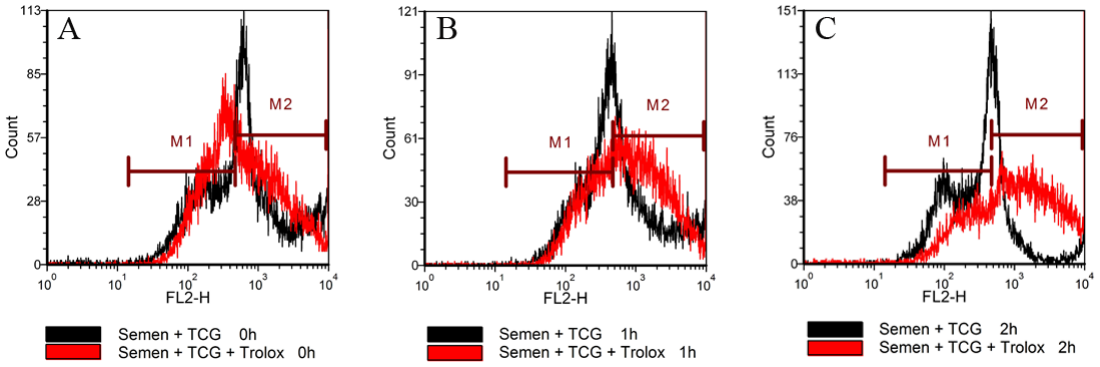
Effect of addition of vitamin E analogue Trolox to TCG extender on sperm mitochondrial membrane potential during incubation at 37°C for 2 h. Mitochondrial membrane potential was detected using JC-1 (lipophilic cation 5,5’,6’,6,- tetrachloro-1,1’,3,3’, -tetraethylbenzimidazolcarbocyanine iodide) Mitochondrial Membrane Potential Detection Kit. Histogram shows spermatozoa with high (marker M2) and low (marker M1) mitochondrial membrane potential.

**Fig 9:**
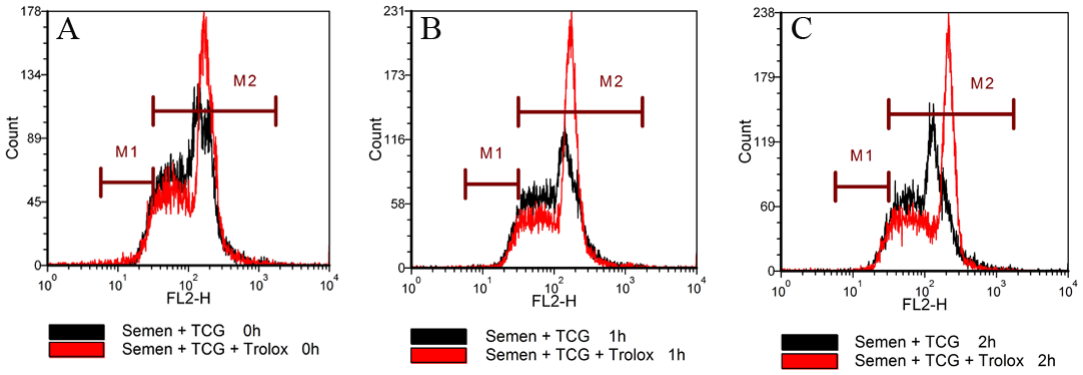
Effect of addition of vitamin E analogue Trolox to TCG extender on lipid peroxidation in spermatozoa during incubation at 37°C for 2 h. Histogram shows spermatozoa with lipid peroxidation (marker M1) and without lipid peroxidation (marker M2).

In order to detect whether vitamin E directly improves sperm quality during incubation as well, fresh semen was diluted with TCG containing 200 μM Trolox and incubated at 37°C for 2 h. As a control, the semen diluted TCG without Trolox and treated as above. The values of motility and intact acrosome were not significantly different between the two groups. The value of membrane integrity in the Trolox group was significantly higher than that of the control group at 2 h of incubation (Fig 7). Interestingly, at 0 h, 1 h and 2 h of incubation, the values of mitochondrial membrane potential in the control group were 54.5%, 46.7% and 32.3%, respectively, while in Trolox group they were 52.8%, 62.5%, 66.8%, respectively (Fig 8), indicating that addition of vitamin E analogue enhanced the mitochondrial membrane potential. On the contrary, LPO values in the Trolox group showed a tendency to decrease compared with the control group (Fig 9).

#### Reactive oxygen species (ROS)

**Fig 10:**
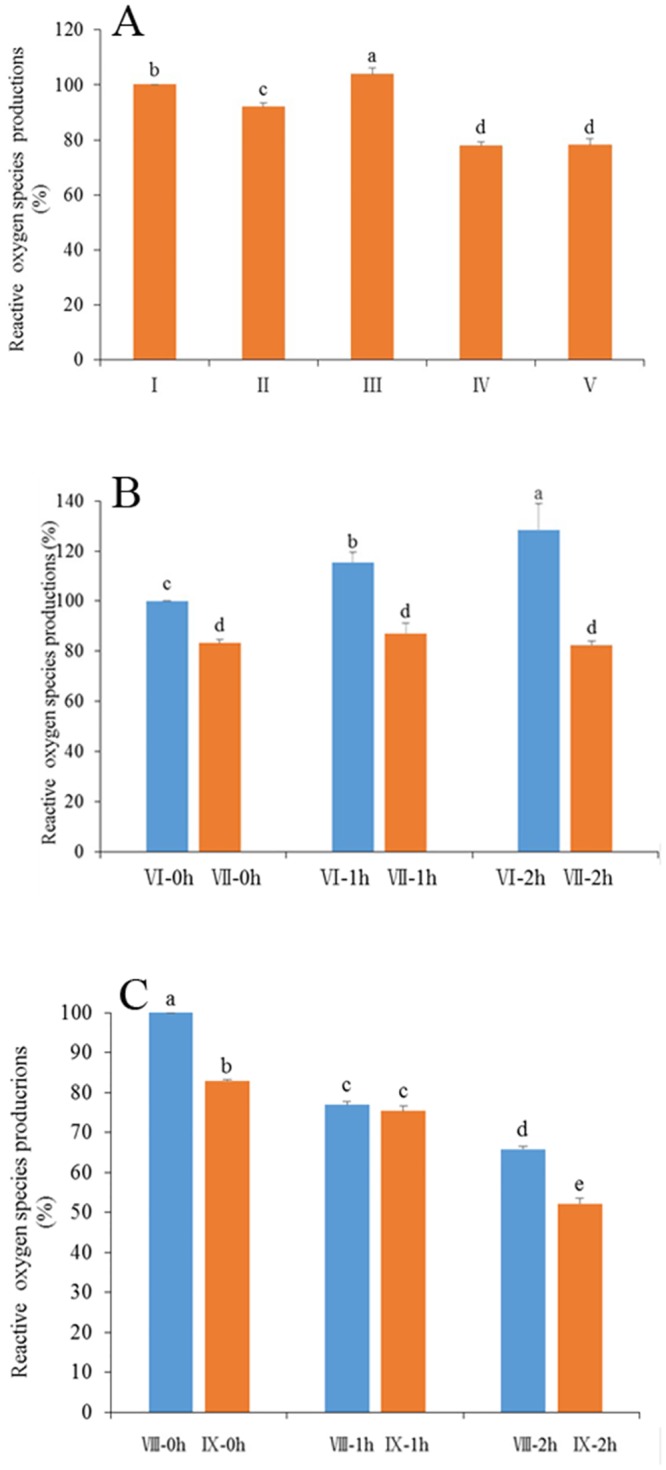
Measure of reactive oxygen species during process of preservation. Bars represent the mean ± SEM (n = 3 independent replicates). Different lower-case letters denote significant differences (P < 0.05). I freshly ejaculated spermatozoa diluted with TCG extender cooling from room temperature to 5°C; II freshly ejaculated spermatozoa diluted with TCG extender containing 200 μM vitamin E analogue Trolox cooling from room temperature to 5°C; III freshly ejaculated spermatozoa diluted with TCG extender cooling from room temperature to 5°C and then mixed with freezing extender and equilibrated for 30 min at 5°C; IV freshly ejaculated spermatozoa diluted with TCG extender cooling from room temperature to 5°C and then mixed with freezing extender containing 200 μM Trolox and equilibrated for 30 min at 5°C; V freshly ejaculated spermatozoa diluted with TCG extender with 200 μM Trolox cooling from room temperature to 5°Cand then mixed with freezing extender containing 200 μM Trolox and equilibrated for 30 min at 5°C; VI the frozen-thawed spermatozoa that were frozen in freezing extender without 200 μM Trolox, thawed in thawing solution without Trolox and incubated at 37°C for 2 h (VI-0h, VI-1h, VI-2h); VII the frozen-thawed spermatozoa that were frozen in freezing extender with 200 μM Trolox, thawed in thawing solution and incubated at 37°C for 2 h (VII-0h, VII-1h, VII-2h); VIII freshly ejaculated spermatozoa frozen in freezing extender without Trolox, thawed in thawing solution with 200 μM Trolox and incubated at 37°C for 2 h (VIII-0h, VIII-1h, VIII-2h); IX freshly ejaculated spermatozoa frozen in freezing extender with 200 μM Trolox, thawed in thawing solution with 200 μM Trolox and incubated at 37°C for 2 h (IX-0h, IX-1h, IX-2h).

To elucidate the mechanism by which vitamin E analogue improves the parameters of frozen-thawed spermatozoa, sperm ROS level was measured. As shown in Fig 10A, when fresh semen was diluted with TCG extender at room temperature and cooled to 5°C, supplementation TCG with 200 μM of Trolox significantly decreased (P < 0.05) ROS accumulation during the cooling process compared with the control (100% vs 92.0%). When the cooled semen was furthermore mixed with freezing extender and equilibrated for 30 min at 5°C, ROS in the equilibrated spermatozoa was significantly accumulated compared with that before equilibration (P < 0.05). Interestingly, ROS level in the spermatozoa mixed with freezing extender containing 200 μM Trolox was significantly lower than that of the control (P < 0.05) at the end of equilibration. The ROS level in spermatozoa exposed to Trolox during both cooling and equilibration was similar compared with that exposed to Trolox only during equilibration. These observations suggest that ROS in spermatozoa was accumulated during both cooling and equilibration, and that addition of the vitamin E analogue quenched the ROS.

To elucidate whether addition of vitamin E protects spermatozoa from ROS stress during freezing-thawing and incubation in vitro after thawing, Trolox was added in freezing extender and thawing solution at the concentration of 200 μM. At 0 h post-thaw, spermatozoa frozen in freezing extender containing Trolox showed lower ROS level (P < 0.05) than that of the control (Fig 10B), suggesting that addition the vitamin E analogue prevent ROS accumulation during process of freezing and thawing. ROS level in the control increased by the time during incubation in vitro (P < 0.05), while it was similar in spermatozoa frozen in freezing extender with Trolox (Fig 10B). Supplementation of Trolox to the extenders quelled ROS accumulation in spermatozoa during freezing-thawing and incubation in vitro (Fig 10C). The ROS level in the frozen-thawed spermatozoa thawed in thawing solution with Trolox was significantly lower than that of spermatozoa thawed in thawing solution without Trolox during post-thaw incubation in vitro (Fig 10C). These observations suggest that ROS in spermatozoa was accumulated during the process of freezing-thawing and post-thaw incubation in vitro, and that addition of the vitamin E analogue quelled the ROS.

## Discussion

The objective of the present study was to determine whether addition of vitamin E to freezing extender could improve the quality of frozen-thawed spermatozoa in rabbits, and how vitamin E protects spermatozoa against damages during the process of preservation. In this study, it was found that supplementation of vitamin E analogue with freezing extenders at a concentration of 200 μM significantly improved post-thaw motility, intact acrosome, membrane integrity and mitochondrial membrane potentials. It was clear that ROS accumulated during the process from cooling, equilibration, freezing-thawing to post-thaw incubation, and that the vitamin E analogue protected spermatozoa from damages during the process of preservation through its ability to quench ROS accumulation.

When the oxidants exceed the antioxidants, an imbalance exists between ROS production and the biological system’s ability to readily detoxify the reactive intermediates, and thereby resulting in damage which is known as oxidative stress [31]. When ROS has been accumulated beyond the level out of the antioxidant system of spermatozoa, sperm motility declined [32]. Free radical causes LPO which is accompanied by extensive structural alterations, particularly in the acrosomal region, a rapid and irreversible loss of motility, a profound change in metabolism, and a high rate of leakage of intracellular cell constituents [5].

Sperm membranes are very rich in long-chain polyunsaturated fatty acids (PUFA) [33]. The double bonds in PUFA molecules make the plasma membrane vulnerable to the attack of free radicals and initiation of LPO cascades. Although the ejaculated spermatozoa are protected against oxidative stress because of the presence of antioxidant defense systems within the spermatozoa and in the seminal plasma [32, 34], the antioxidant capability in spermatozoa is very limited as most of their cytoplasmic components, which contain sufficient antioxidants to counteract the harmful effects of ROS and LPO, and is lost during spermiogenesis. Furthermore, dilution of semen further yields less antioxidants available for the spermatozoa. Therefore, spermatozoa are sensitive to oxidative stress during the process of preservation. Cryopreservation significantly reduced the levels of antioxidant defenses in spermatozoa [34] and generated ROS [34–36]. As the endogenous antioxidant capacity may be insufficient to prevent ROS and LPO, supplementation of antioxidants with cryopreservation extenders would be beneficial to spermatozoa.

Spermatozoa are highly susceptible to LPO. The spontaneous membrane LPO destroys the structure of the lipid matrix by attacks from ROS. ROS ultimately lead to loss of sperm function, such as functional membrane integrity, sperm motility and fertility potential [35–36]. Vitamin E is believed to be the primary component of the antioxidant system of spermatozoa, and is regarded as one of the major membrane protectants against ROS and LPO [37–39]. In the present study, we found that exposure of spermatozoa to the vitamin E analogue Trolox significantly resulted in less ROS accumulation during the whole process of cryopreservation, including cooling from room temperature to 5°C, equilibrating, freezing-thawing, and incubation in vitro after thawing (Fig 10). Meanwhile, exposure to Trolox led to less LPO in the frozen-thawed spermatozoa (Fig 9) as well.

The decreased ΔΨm is a sensitive indicator of mitochondrial damage, which was assessed by measuring cellular retention of the fluorescent probe of JC-1. The mean red fluorescent intensity in the vitamin E treated post-thaw spermatozoa was significantly higher than that of the control (Fig 3), indicating that incubation with vitamin E analogue caused a marked increase in the mitochondrial membrane potential of rabbit spermatozoa.

Plasma membrane integrity and intact acrosome are the main parameters in the evaluation of sperm functionality [40]. In the present study, supplementation of 200 μM or 250 μM of vitamin E analogue with the freezing extender resulted in significant increase of the percentage of post-thaw spermatozoa with intact acrosome which is in agreement with the findings of Hu et al. [41] who demonstrated that adding vitamin E to freezing-medium improved the percentages of intact acrosomes in bulls. Acrosome damaged may be caused during cryopreservation in which spermatozoa encounter stresses such as oxidative, thermal and osmotic stresses [42]. Similarly, addition of vitamin E analogue protects the plasma membrane from cryodamage as well (Fig 1C), which is consistent with the findings in rams [18], boars [7, 43] and bulls [39]. These observations indicated that vitamin E addition prior to freezing provides greater structural integrity for rabbit spermatozoa after cryopreservation.

In this study, post-thaw rabbit sperm motility was significantly improved by the addition of 200 μM Trolox which is in agreement with the previous reports in boars [10] and humans [14]. Breininger et al. [8] and Pena et al. [4, 43] found that supplementation of vitamin E with the cryopreservation media prior to cryopreservation significantly enhanced survival rate of post-thaw boar spermatozoa. Taylor et al. [14] showed the dose of vitamin E was significantly associated with post-thaw human sperm motility.

## Conclusion

Taken together, the addition of vitamin E analogue to a freezing extender leads to higher rates of integrity of the plasma membrane, acrosome and mitochondrial membrane potential as well as higher motility in frozen-thawed rabbit spermatozoa. Vitamin E protects spermatozoa through its capacity to quench ROS accumulation and LPO during the process of preservation.
